# Pathogenicity Reclassification of *RPE65* Missense Variants Related to Leber Congenital Amaurosis and Early-Onset Retinal Dystrophy

**DOI:** 10.3390/genes11010024

**Published:** 2019-12-24

**Authors:** Fabiana L. Motta, Renan P. Martin, Fernanda B. O. Porto, Elizabeth S. Wohler, Rosane G. Resende, Caio P. Gomes, João B. Pesquero, Juliana M. F. Sallum

**Affiliations:** 1 Department of Ophthalmology, Universidade Federal de São Paulo, Sao Paulo SP 04039-032, Brazil; fabiana.louise@gmail.com; 2 Instituto de Genética Ocular, Sao Paulo SP 04552-050, Brazil; 3 McKusick-Nathans Department of Genetic Medicine, Johns Hopkins Medicine, Baltimore, MD 21205, USA; renan.paulo.martin@gmail.com (R.P.M.); esquibb1@jhmi.edu (E.S.W.); 4 INRET Clínica e Centro de Pesquisa, Belo Horizonte MG 30150-270, Brazil; fernandabop@gmail.com; 5 Centro Oftalmológico de Minas Gerais, Belo Horizonte MG 30180-070, Brazil; 6 Instituto de Olhos Carioca, Rio de Janeiro RJ 22220-080, Brazil; roguaziresende@gmail.com; 7 Department of Biophysics, Universidade Federal de São Paulo, São Paulo SP 04039-032, Brazil; caiopgomes@hotmail.com (C.P.G.); jbpesquero@gmail.com (J.B.P.)

**Keywords:** *RPE65* gene, variant of uncertain significance (VUS), likely pathogenic variant, Leber congenital amaurosis (LCA), early-onset retinal dystrophy (EORD)

## Abstract

A challenge in molecular diagnosis and genetic counseling is the interpretation of variants of uncertain significance. Proper pathogenicity classification of new variants is important for the conclusion of molecular diagnosis and the medical management of patient treatments. The purpose of this study was to reclassify two *RPE65* missense variants, c.247T>C (p.Phe83Leu) and c.560G>A (p.Gly187Glu), found in Brazilian families. To achieve this aim, we reviewed the sequencing data of a 224-gene retinopathy panel from 556 patients (513 families) with inherited retinal dystrophies. Five patients with p.Phe83Leu and seven with p.Gly187Glu were selected and their families investigated. To comprehend the pathogenicity of these variants, we evaluated them based on the American College of Medical Genetics and Genomics and the Association for Molecular Pathology (ACMG/AMP) classification guidelines. Initially, these *RPE65* variants met only three pathogenic criteria: (i) absence or low frequency in the population, (ii) several missense pathogenic *RPE65* variants, and (iii) 15 out of 16 lines of computational evidence supporting them as damaging, which together allowed the variants to be classified as uncertain significance. Two other pieces of evidence were accepted after further analysis of these Brazilian families: (i) p.Phe83Leu and p.Gly187Glu segregate with childhood retinal dystrophy within families, and (ii) their prevalence in Leber congenital amaurosis (LCA)/early-onset retinal dystrophy (EORD) patients can be considered higher than in other inherited retinal dystrophy patients. Therefore, these variants can now be classified as likely pathogenic according to ACMG/AMP classification guidelines.

## 1. Introduction

Currently, the molecular diagnosis of rare genetic diseases with clinical and genetic heterogeneity, such as inherited retinal dystrophies (IRD) [1,2], has become more accessible and accurate with the use of next-generation sequencing [3,4,5,6]. However, the interpretation of variants found in these molecular tests is still a major challenge for all professionals involved in molecular diagnosis and genetic counseling. Typically, novel variants have never been previously described and their impacts on the gene or protein are unknown; therefore, they are classified as variants of uncertain significance (VUS), rendering the genetic diagnosis inconclusive.

The American College of Medical Genetics and Genomics and the Association for Molecular Pathology (ACMG/AMP) guidelines [7] suggest that genetic variants are classified on a five-level scale: pathogenic, likely pathogenic, uncertain significance, likely benign, and benign. For this classification to be made, different criteria must be considered together for an assessment of the likelihood of any variant being pathogenic or benign.

Briefly, the criteria for classifying the pathogenicity of variants are: (i) variants that have been reported to cause disease in the literature, (ii) variants that lead to loss of protein function, (iii) variants that are absent or infrequent in genetic variant databases, (iv) variants not described, but present in other affected patients, and (v) variants that have not been reported in the literature, but are predicted by in silico analysis as likely damaging. Further description of variant classification guidelines is detailed in the ACMG/AMP guidelines [7].

Several pathogenic variants have already been described in the *RPE65* gene [8,9,10] which lead to childhood retinal dystrophy, ranging from Leber congenital amaurosis (LCA) to early-onset retinal dystrophy (EORD). These diseases are the earliest and most severe inherited retinal dystrophies [11,12]. Appropriate pathogenicity classification of novel variants is relevant not only to the conclusion of molecular diagnosis and genetic counseling but also to medical management of the condition. Currently, a gene therapy treatment is available for *RPE65* (LUXTURNA^®^-voretigene neparvovec-rzyl). For these reasons, this paper aims to reclassify two of the most frequent *RPE65* missense variants of uncertain significance found in Brazilian families.

## 2. Materials and Methods

This retrospective cross-sectional study was approved by the Ethics Committee in Research of Universidade Federal de São Paulo (CEP: 0415/2016). Written informed consent for the use of personal medical data for scientific purposes and publication was obtained from all patients. In addition, this study was performed in accordance with the ethical standards of the 1964 Declaration of Helsinki and its subsequent amendments.

### 2.1. Subjects

Five hundred and fifty-six retinopathy gene panels from IRD patients (513 families), recruited from five specialized services in inherited retinal dystrophy in Brazil (Universidade Federal de São Paulo, Instituto de Genética Ocular, INRET Clínica e Centro de Pesquisa, Centro Oftalmológico de Minas Gerais, and Instituto de Olhos Carioca), were reviewed in order to find patients with c.247T>C (p.Phe83Leu) or c.560G>A (p.Gly187Glu) variants in *RPE65* (NM_000329.3). All genetic data analyzed were based on sequencing from a 224-gene retinopathy panel (Supplementary). The p.Phe83Leu variant was found exclusively in five homozygous patients from four families, while p.Gly187Glu was present only in seven homozygous patients from three families. Neither p.Phe83Leu nor p.Gly187Glu was found in other IRD probands from our sample. Medical and family histories of these 12 patients were collected. Most patients were ophthalmologically evaluated. Clinical diagnosis was based on signs/symptoms, ophthalmologic features, and age of onset.

These two variants were chosen for further analysis because among 513 Brazilian families analyzed with IRD history, 15 potentially disease-causing variants of *RPE65* were found. Five of them were missenses and considered pathogenic in the literature: p.Leu22Pro [13,14,15,16,17,18], p.Arg91Pro [19], p.Arg91Gln [16,17,20,21,22], p.Leu341Ser [22,23,24], and p.Gly528Val [20,25]. Only p.Phe83Leu and p.Gly187Glu were variants of uncertain significance. Additionally, these variants were present in more than one family and in at least one family with more than one affected member. Outside of these two variants, no additional missense biallelic variants were found among the 513 analyzed families.

### 2.2. Family Segregation

**Figure 1:**
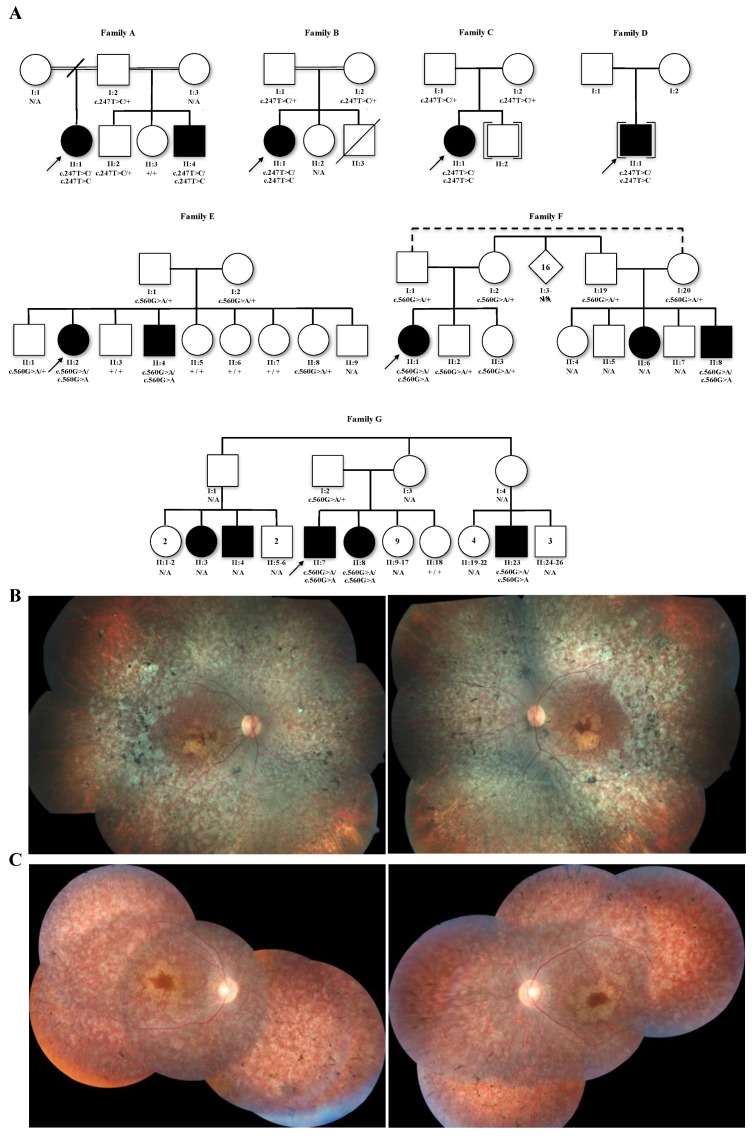
Pedigrees and clinical findings of *RPE65* patients. (**A**) Pedigrees of c.247T>C (p.Phe83Leu) families with LCA (families A to D) and c.560G>A (p.Gly187Glu) families with LCA/EORD (families E to G). All probands and some affected relatives genotyped are homozygotes. (**B**) Color fundus photograph of a proband at age 37 from family B. (**C**) Color fundus photograph of a proband at age 35 from family F. N/A—data not available.

In order to verify if the disease segregated only with homozygous variants, segregation analysis was performed in all available probands’ informative relatives (Figure 1A). Sanger sequencing was performed using genomic DNA from whole blood cells and specific oligonucleotide primers flanking exons 4 and 5 to detect p.Phe83Leu (5′-GGC CAT TCT AAG CTC CAC ATG-3′ and 5′-GCA GGA AAG CAT CAT AGA CAG AG-3′), and flanking exon 6 to detect p.Gly187Glu (5′-TCC AGA TTC AGA CAC ATG AGG-3′ and 5′-TAG GGT AGG GAT GAG GGC AG-3′).

### 2.3. Variant Analysis

The evaluation of the p.Phe83Leu and p.Gly187Glu variants in *RPE65* was made according to ACMG standardization [7]. For proper classification of pathogenicity, population databases (The Genome Aggregation Database (gnomAD) [26], The Exome Aggregation Consortium (ExAC) [27], The NHLBI GO Exome Sequencing Project (ESP) [28], the 1000 Genomes Project [29], Online Archive of Brazilian Mutations (ABraOM) [30]) and human variation and phenotype databases (ClinVar) [8], The Universal Protein Resource (UniProt) [9], and The Human Gene Mutation Database (HGMD^®^) [10]) were consulted. In addition, variants were evaluated by multiple in silico predictive algorithms (Polymorphism Phenotyping v2 (PolyPhen2) [31], Sorting Intolerant From Tolerant (SIFT) [32], Deleterious Annotation of genetic variants using Neural Networks (DANN) [33], Protein Variation Effect Analyzer (PROVEAN) [34], the Likelihood Ratio Test (LRT) [35], MutationTaster [36], MutationAssessor [37], Functional Analysis through Hidden Markov Models (FATHMM) [38], Functional Analysis through Hidden Markov Models—Multiple Kernel Learning (FATHMM-MKL) [39], Mendelian Clinically Applicable Pathogenicity (M-CAP) [40], Combined Annotation-Dependent Depletion (CADD) [41], Ensembl Meta-analytic Support Vector Machine (MetaSVM) [42], Ensembl Meta Logistic Regression (MetaLR) [43], Phylogenetic *P*-values to multiple alignments of 20 species (PhyloP 20way) [44], Phylogenetic *P*-values to multiple alignments of 100 species (PhyloP 100way) [44], and The Genomic Evolutionary Rate Profiling—Rejected Substitution (GERP RS) [45]) that analyze amino acid conservation and/or the putative impact of variants on protein structure or functionality. In order to assess the association between childhood retinal dystrophies and *RPE65* variants, the odds ratios (OR) of LCA/EORD with Haldane–Anscombe correction and 97.5% confidence intervals (corrected by Bonferroni multiple-comparison correction) were calculated using IBM SPSS Statistics. An OR ≥ 3, with a confidence interval which did not include the number 1, was considered as a positive association between the found variant and the disease.

## 3. Results and Discussion

### 3.1. Pathogenicity Classification of RPE65 Variants c.247T>C (p.Phe83Leu) and c.560G>A (p.Gly187Glu) According to the Current Literature and Databases

Missense variants related to recessive diseases should be classified for pathogenicity following certain criteria/evidence established by the ACMG/AMP. Each piece of evidence is weighted as very strong, strong, moderate, or supporting [7]. Some criteria related to dominant disease as well as null variants were not addressed here, because the genic variants under consideration are of the missense type and the gene is related only to recessive diseases. Another criterion not discussed was whether the phenotype is highly specific for a disorder with a single disease-causing gene because inherited retinal dystrophies have significant genetic heterogeneity and relevant clinical overlap.

Two studies have already reported these variants, but they did not confirm or discuss the pathogenicity of c.247T>C (p.Phe83Leu) or c.560G>A (p.Gly187Glu) [22,46]; moreover, these two reported cases are Brazilian and are included in this study (family B and E probands). Based on current knowledge of these two *RPE65* variants, no strong evidence has been identified, as there are no previously reported mutations which, regardless of nucleotide change, result in p.Phe83Leu or p.Gly187Glu, and no functional studies showing that these variants cause deleterious effects. 

Two other moderate criteria that evaluate the position where the new missense mutation occurs were also not included: (i) outside of these two variants already reported [22,46], no other damaging missense variant in the amino acid residue Phe83 or Gly187 has been reported before and, (ii) the protein region where both variants occur is neither a mutational hot spot nor a well-established functional domain.

Both variants in this study have already been associated with rare recessive retinal dystrophies, each of which was identified in only one homozygous patient [22,46]. Therefore, the criterion regarding the identification of evaluated variants in trans with known pathogenic mutations was not met (ACMG/AMP pathogenic moderate level of evidence). The variants evaluated here are rare: c.247T>C; p.Phe83Leu is a novel variant not found in any of the five population databases queried (Table 1), and c.560G>A; p.Gly187Glu was only seen twice (two heterozygous individuals) out of more than 125,000 individuals analyzed (Table 1) (ACMG/AMP moderate criterion). Based on the current literature and their extremely low frequencies, it cannot be claimed that these variants were significantly more frequent in affected people than either in the controls or as was observed in many unrelated patients (ACMG/AMP pathogenic strong/moderate criterion), nor that they segregated with disease in several affected family members (ACMG/AMP supporting evidence criterion).

The ClinVar [8] and UniProt [9] databases show that there are 69 pathogenic variants in the *RPE65* gene, of which 52 are missenses. While HGMD [10] lists 191 *RPE65* disease-causing mutations, 103 of these are missense variants, which suggests that missense changes are a common cause of *RPE65*-associated IRD (ACMG/AMP supporting evidence criterion). In addition, 15 out of 16 in silico prediction tools support that the amino acids of both variants are conserved and/or that their changes would negatively impact protein function (Table 2) (ACMG/AMP supporting evidence criterion).

Initially, the c.247T>C; p.Phe83Leu and c.560G>A; p.Gly187Glu variants met only three pieces of ACMG/AMP pathogenic evidence: (i) absence or low frequency in the population (ACMG/AMP pathogenic moderate criterion PM2), (ii) *RPE65* having many pathogenic missense variants (ACMG/AMP supporting level of evidence PP2), and (iii) several lines of computational prediction evidence supporting them as disease-causing mutations (ACMG/AMP supporting criterion PP3). Based on the combination of these three criteria, these variants were classified as variants of uncertain significance.

### 3.2. Evaluation of the RPE65 Variants p.Phe83Leu and p.Gly187Glu in Brazilian Families with Inherited Retinal Dystrophies

The p.Phe83Leu alleles were found in five homozygous LCA patients from four unrelated families (families A to D; Figure 1A), while the p.Gly187Glu alleles were identified in seven LCA/EORD homozygous patients from three different families. Some of these families had other affected individuals who were not accessible for genetic testing (families E to G; Figure 1A). Segregation analysis in several family members showed that retinal dystrophy segregates together with the homozygous genotype of these two variants. All heterozygous or wild-type individuals for their respective family mutations were unaffected (Figure 1A).

All affected individuals with the genotype of homozygous p.Phe83Leu were diagnosed with LCA. Nystagmus was a common clinical finding, and the onset of the first signs and symptoms, such as low vision and/or nystagmus, occurred before 1 year of age (Table 3). The clinical diagnosis for p.Gly187Glu patients was mostly EORD, and nystagmus was not reported by all patients. The typical features of the *RPE65* fundus [22] found in these patients were pigmentary changes (granular appearance of retinal pigment epithelium and peripheral bone spicule pigmentation) that progress to areas of atrophy. In addition, in more advanced cases, macular atrophic changes were observed (Figure 1B,C), and some patients also had vessel attenuation (Figure 1C).

The allelic frequency of the variants p.Phe83Leu and p.Gly187Glu in Brazilian probands with inherited retinal dystrophies was 0.0078 (8 of 1026 alleles) and 0.0058 (6 of 1026 alleles), respectively (Table 4). No heterozygous or compound heterozygous variants were identified in these samples, which means that p.Phe83Leu and p.Gly187Glu variants were only found in the cases exposed in this study. 

Table 5 presents the data used to compare the prevalence of homozygous variants in patients with LCA/EORD and other IRD patients. The calculated odds ratios of LCA/EORD (OR_Phe83Leu_ = 42.285; 97.5% CI_Phe83Leu_ = (1.482, 1206.268) and OR_Gly187Glu_ = 32.525; 97.5% CI_Gly187Glu_ = (1.088, 972.642)) indicate a positive association between LCA/EORD and homozygous p.Phe83Leu and p.Gly187Glu variants, suggesting a higher prevalence of these variants in individuals with LCA/EORD than with other subtypes of IRD.

These findings in Brazilian patients suggest the homozygous variants, p.Phe83Leu and p.Gly187Glu, apparently segregate together with childhood retinal dystrophy within families (ACMG/AMP pathogenic supporting criterion PP1). In addition, these variants were observed in several unrelated patients with similar phenotypes and were positively associated with LCA/EORD, presenting odds ratios greater than 3 and 97.5% confidence intervals not including 1.00. Therefore, the prevalence of p.Phe83Leu and p.Gly187Glu in individuals affected by LCA/EORD can be considered significantly higher compared with other inherited retinal dystrophies (ACMG/AMP pathogenic strong level of evidence PS4).

This study adds two more pieces of evidence of pathogenicity in the evaluation of the *RPE65* variants p.Phe83Leu and p.Gly187Glu, one supporting (PP1) and one strong (PS4). Thus, these variants meet five ACMG/AMP pathogenicity criteria, one strong (PS4), one moderate (PM2) and three supporting (PP1, PP2, and PP3), which allows us to reclassify them as likely pathogenic [7]. Some criteria proposed by the ACMG/AMP are for cases of dominant diseases, variants with premature stop codon formation, or for syndromic cases. Thus, little evidence of pathogenicity remained to be evaluated in this study.

Confirmation of one more additional criterion, presented below, would change the classification to pathogenic. The presence of compound heterozygote affected individuals, with variants in trans where one variant is known to be pathogenic, would allow us to accept the moderate criterion PM3. The PP1 criteria could be improved from a supporting criterion to either moderate or strong if more families of different ethnicities present the same variant. The addition of either of these criteria would lift the final classification from likely pathogenic to pathogenic. 

The PP4 criterion, accounting for the use of phenotype to support the variant, could not be fully met because the ACMG/AMP guidelines specify four criteria, in which only three of them are met: (i) the clinical sensitivity of testing to diagnose affected individuals is high; (ii) the gene is not subject to substantial benign variation—according to the Varsome database [47] only eight variants are classified as benign out of 93 present in their database (version November 28, 2019); and (iii) family history is consistent with the mode of inheritance of the disorder—in this sample group all affected individuals have homozygous variants and all heterozygous carriers are unaffected. Criteria (iv) could not be utilized because LCA and EORD are not syndromic.

The ACMG/AMP guidelines are a useful tool for increasing the concordance of variant interpretations. However, it is noted that some criteria proposed by these guidelines are too restrictive (e.g., PP4), questionable (e.g., PM3 and PP5), or the way of applying it is not very clear (e.g., PP1). Thus, the identification of a rare variant exclusively in individuals affected by the same disease from different families is a relevant criterion to evaluate the pathogenicity of the variant. 

Initial classification of these two homozygous variants determined they were variants of uncertain significance. However, including a systematic review of mutations present in a population sample allowed for their reclassification to likely pathogenic. Identifying variants with a greater potential for pathogenicity enables researchers to prioritize those variants for further evaluation and exploration by in vivo or in vitro functional studies.

Several animal models for inherited retinal dystrophy used for in vivo functional studies have been reported, including dog and rodent models [48,49,50,51]. Animals with *RPE65* variants are able to develop human-like retinopathies, allowing investigators to evaluate the effect of these variants during retinal development and even in advanced stages of animal life in order to establish a more robust genotype–phenotype correlation [52,53,54,55,56,57]. Studies have shown that *RPE65* mutations can modulate disease phenotypes, and variants in other genes can also influence *RPE65*-related phenotype [58,59,60,61]. In this respect, animal models are also informative as they allow the creation of specific genotypes to assess the effect of variant combinations on phenotype severity. The evaluation of gene therapy treatments in animal models is invaluable as it allows for the evaluation of potential outcomes or complications of such therapies [62,63,64,65,66,67]. 

Despite the many advantages of functional studies with animal models, this strategy is often expensive, laborious, and can take significant time before the first genetically modified organisms can be evaluated. To overcome these obstacles, in vitro studies evaluating the correct production and expression of the RPE65 protein (e.g., immunoblot, RT-qPCR, and RNA-sequencing), as well as its enzymatic activity, are advantageous. The evaluation of isomerhydrolase activity of *RPE65* can be measured using iPS-derived retinal pigment epithelium (RPE) cells from patients, hES-derived RPE cells modified by site-directed mutagenesis, or transfected cells with a mutated *RPE65* expression vector [14,25,68,69].

Recently, Li and colleagues (2019) presented new perspectives for personalized functional studies utilizing retinal organoids containing RPE and photoreceptor cells derived from human-induced pluripotent stem cells [69]. Despite the need for further studies to evaluate the ability of these organoids to mimic the phenotype and molecular aspects of the retina, the generation of these organoids can still enhance the study of cell replacement therapies.

In regards to gene therapy, Yang et al. (2019) also discussed the need for in vitro analysis to verify the pathogenicity of VUS variants prior to the administration of voretigene neparvovec-rzyl, thus avoiding treatments of false-positive individuals [70]. They also highlighted the importance of this process, which will allow clinicians to choose relevant candidates for gene therapy.

## 4. Conclusions

These study findings suggest that the molecular diagnosis of patients with p.Phe83Leu and p.Gly187Glu variants, in homozygosis or in trans with another pathogenic variant in the *RPE65* gene, should be considered likely pathogenic. Therefore, these patients are possible candidates for gene therapy treatments. This approach of revisiting the variant classification status based on descriptions of new affected individuals could help to improve variant classification and consequently have an impact on clinical practice and molecular diagnosis. 

## Figures and Tables

**Table 1:** Evaluation of p.Phe83Leu and p.Gly187Glu in genetics databases.

	c.247T>C; p.Phe83Leu	c.560G>A; p.Gly187Glu
*Population Genetics Databases*		
gnomAD total	No data	2 in 251,106 alleles
ExAC total	No data	1 in 120,880 alleles
ESP	No data	No data
1000 Genomes	No data	No data
ABraOM	No data	No data
*Human Variations and Phenotypes Databases*		
ClinVar	Not reported	Not reported
UniProt	Not reported	Not reported
HGMD	Disease-Causing Mutation [22]	Disease-Causing Mutation [46]

**Table 2:** Evaluation of p.Phe83Leu and p.Gly187Glu by 16 predictors.

	Prediction or Score	
In Silico Prediction Tool	c.247T>C; p.Phe83Leu	c.560G>A; p.Gly187Glu
PolyPhen2	Possibly damaging	Probably damaging
SIFT	Damaging	Damaging
DANN (max. damaging score 1)	0.9955	0.9979
PROVEAN	Damaging	Damaging
LRT	Deleterious	Deleterious
MutationTaster	Disease-causing	Disease-causing
MutationAssessor	Medium impact	Medium impact
FATHMM [38]	Damaging	Damaging
FATHMM-MKL	Damaging	Damaging
M-CAP	Damaging	Damaging
CADD (>20 more likely the SNV is damaging)	24.9	32
MetaSVM	Damaging	Damaging
MetaLR	Damaging	Damaging
PhyloP 20way (max. conservation score 1.199000)	1.199000	0.807000
PhyloP 100way (max. conservation score 10.003000)	8.735000	7.461000
GERP RS (max. conservation score 6.17)	5.03	5.54

**Table 3:** Clinical data of genotyped affected patients with p.Phe83Leu or p.Gly187Glu *RPE65* variant.

Family	Patient	Diagnosis	Age of Onset	Current Age	Visual Acuity (OD;OE)	Other Findings
A	II:1	LCA	since birth	27 years	20/1600; 20/800	Nystagmus, Light-seeking behavior
	II:4	LCA	since birth	20 years	20/200; 20/200	Nystagmus, Light-seeking behavior
B	II:1	LCA	before 1 year	37 years	20/800; 20/1600	Nystagmus
C	II:1	LCA	before 1 year	49 years	Light Perception	Nystagmus, Optic nerve drusen
D	II:1	LCA	before 1 year	13 years	N/A	Nystagmus
E	II:2	EORD	N/A	39 years	20/400; 20/400	Nystagmus
	II:4	EORD	N/A	34 years	N/A	N/A
F	II:1	LCA	since birth	35 years	20/60; 20/60	No nystagmus
	II:8	LCA	since birth	37 years	20/400; 20/200	No nystagmus
G	II:7	EORD	N/A	54 years	20/800; 20/500	N/A
	II:8	EORD	7 years	51 years	Hand Movement	N/A
	II:23	EORD	N/A	71 years	20/500; 20/500	N/A

LCA—Leber congenital amaurosis; EORD—early-onset retinal dystrophy; N/A—not available.

**Table 4:** Allele frequency of *RPE65* variants among Brazilian IRD patients (n = 513 probands).

Nucleotide Change *	Consequence	Brazilian IRD Patients			
		Allele Count	Allele Number	Allele Frequency	Homozygotes
c.247T>C	p.Phe83Leu	8	1026	0.0078	4
c.560G>A	p.Gly187Glu	6	1026	0.0058	3

* based on transcript NM_000329.3; IRD—inherited retinal dystrophy.

**Table 5:** Cross-sectional study with 556 Brazilian IRD patients from 513 families.

Genotype	LCA/EORD (*n* = 93)	Other IRD (*n* = 420)	OR * (97.5%CI) †
c.247T>C;p.Phe83Leu Homozygous	4	0	42.285 (1.482–206.268)
c.247T>C;p.Phe83Leu Non-Homozygous	89	420	
c.560G>A;p.Gly187Glu Homozygous	3	0	32.525 (1.088–972.642)
c.560G>A;p.Gly187Glu Non-Homozygous	90	420	

* OR—odds ratio with Haldane–Anscombe correction; † confidence interval of 97.5% with Bonferroni multiple-comparison correction; LCA—Leber congenital amaurosis; EORD—early-onset retinal dystrophy; IRD—inherited retinal dystrophy.

## Supplementary Materials

The following are available online at https://www.mdpi.com/2073-4425/11/1/24/s1; Genes targeted on retinopathy NGS panel.

## References

[1] Berger W., Kloeckener-Gruissem B., Neidhardt J. (2010). The molecular basis of human retinal and vitreoretinal diseases. Prog. Retin. Eye Res..

[2] Motta F.L., Martin R.P., Filippelli-Silva R., Salles M.V., Sallum J.M.F. (2018). Relative frequency of inherited retinal dystrophies in Brazil. Sci. Rep..

[3] Daiger S.P., Sullivan L.S., Bowne S.J., Birch D.G., Heckenlively J.R., Pierce E.A., Weinstock G.M. (2010). Targeted high-throughput DNA sequencing for gene discovery in retinitis pigmentosa. Adv. Exp. Med. Biol..

[4] Audo I., Bujakowska K.M., Léveillard T., Mohand-Saïd S., Lancelot M.-E., Germain A., Antonio A., Michiels C., Saraiva J.-P., Letexier M. (2012). Development and application of a next-generation-sequencing (NGS) approach to detect known and novel gene defects underlying retinal diseases. Orphanet J. Rare Dis..

[5] Chiang J.P., Trzupek K. (2015). The current status of molecular diagnosis of inherited retinal dystrophies. Curr. Opin. Ophthalmol..

[6] Ellingford J.M., Barton S., Bhaskar S., Williams S.G., Sergouniotis P.I., O’Sullivan J., Lamb J.A., Perveen R., Hall G., Newman W.G. (2016). Whole Genome Sequencing Increases Molecular Diagnostic Yield Compared with Current Diagnostic Testing for Inherited Retinal Disease. Ophthalmology.

[7] Richards S., Aziz N., Bale S., Bick D., Das S., Gastier-Foster J., Grody W.W., Hegde M., Lyon E., Spector E. (2015). Standards and guidelines for the interpretation of sequence variants: A joint consensus recommendation of the American College of Medical Genetics and Genomics and the Association for Molecular Pathology. Genet. Med..

[8] Landrum M.J., Lee J.M., Benson M., Brown G.R., Chao C., Chitipiralla S., Gu B., Hart J., Hoffman D., Jang W. (2018). ClinVar: Improving access to variant interpretations and supporting evidence. Nucleic Acids Res..

[9] The UniProt Consortium (2019). UniProt: A worldwide hub of protein knowledge. Nucleic Acids Res..

[10] Stenson P.D., Mort M., Ball E.V., Evans K., Hayden M., Heywood S., Hussain M., Phillips A.D., Cooper D.N. (2017). The Human Gene Mutation Database: Towards a comprehensive repository of inherited mutation data for medical research, genetic diagnosis and next-generation sequencing studies. Hum. Genet..

[11] Foxman S.G., Heckenlively J.R., Bateman J.B., Wirtschafter J.D. (1985). Classification of Congenital and Early Onset Retinitis Pigmentosa. Arch. Ophthalmol..

[12] Weleber R.G., Francis P.J., Trzupek K.M., Beattie C., Adam M., Ardinger H., Pagon R.E., Wallace S., Bean L., Stephens K., Amemiya A. (2004). Leber Congenital Amaurosis. GeneReviews®.

[13] Marlhens F., Griffoin J.M., Bareil C., Arnaud B., Claustres M., Hamel C.P. (1998). Autosomal recessive retinal dystrophy associated with two novel mutations in the RPE65 gene. Eur. J. Hum. Genet..

[14] Lorenz B., Poliakov E., Schambeck M., Friedburg C., Preising M.N., Redmond T.M. (2008). A Comprehensive Clinical and Biochemical Functional Study of a Novel *RPE65* Hypomorphic Mutation. Investig. Opthalmol. Vis. Sci..

[15] Li S., Izumi T., Hu J., Jin H.H., Siddiqui A.-A.A., Jacobson S.G., Bok D., Jin M. (2014). Rescue of enzymatic function for disease-associated RPE65 proteins containing various missense mutations in non-active sites. J. Biol. Chem..

[16] Xiong H.Y., Alipanahi B., Lee L.J., Bretschneider H., Merico D., Yuen R.K.C., Hua Y., Gueroussov S., Najafabadi H.S., Hughes T.R. (2015). RNA splicing. The human splicing code reveals new insights into the genetic determinants of disease. Science.

[17] Huang X.-F., Huang F., Wu K.-C., Wu J., Chen J., Pang C.-P., Lu F., Qu J., Jin Z.-B. (2015). Genotype-phenotype correlation and mutation spectrum in a large cohort of patients with inherited retinal dystrophy revealed by next-generation sequencing. Genet. Med..

[18] Jin M., Li S., Hu J., Jin H.H., Jacobson S.G., Bok D. (2016). Functional Rescue of Retinal Degeneration-Associated Mutant RPE65 Proteins. Adv. Exp. Med. Biol..

[19] Simonelli F., Ziviello C., Testa F., Rossi S., Fazzi E., Bianchi P.E., Fossarello M., Signorini S., Bertone C., Galantuomo S. (2007). Clinical and molecular genetics of Leber’s congenital amaurosis: A multicenter study of Italian patients. Investig. Ophthalmol. Vis. Sci..

[20] Thompson D.A., Gyürüs P., Fleischer L.L., Bingham E.L., McHenry C.L., Apfelstedt-Sylla E., Zrenner E., Lorenz B., Richards J.E., Jacobson S.G. (2000). Genetics and phenotypes of RPE65 mutations in inherited retinal degeneration. Investig. Ophthalmol. Vis. Sci..

[21] Philp A.R., Jin M., Li S., Schindler E.I., Iannaccone A., Lam B.L., Weleber R.G., Fishman G.A., Jacobson S.G., Mullins R.F. (2009). Predicting the pathogenicity of RPE65 mutations. Hum. Mutat..

[22] Chung D.C., Bertelsen M., Lorenz B., Pennesi M.E., Leroy B.P., Hamel C.P., Pierce E., Sallum J., Larsen M., Stieger K. (2019). The Natural History of Inherited Retinal Dystrophy Due to Biallelic Mutations in the RPE65 Gene. Am. J. Ophthalmol..

[23] Morimura H., Fishman G.A., Grover S.A., Fulton A.B., Berson E.L., Dryja T.P. (1998). Mutations in the RPE65 gene in patients with autosomal recessive retinitis pigmentosa or leber congenital amaurosis. Proc. Natl. Acad. Sci. USA.

[24] Maguire A.M., High K.A., Auricchio A., Wright J.F., Pierce E.A., Testa F., Mingozzi F., Bennicelli J.L., Ying G., Rossi S. (2009). Age-dependent effects of RPE65 gene therapy for Leber’s congenital amaurosis: A phase 1 dose-escalation trial. Lancet.

[25] Redmond T.M., Poliakov E., Yu S., Tsai J.-Y., Lu Z., Gentleman S. (2005). Mutation of key residues of RPE65 abolishes its enzymatic role as isomerohydrolase in the visual cycle. Proc. Natl. Acad. Sci. USA.

[26] Karczewski K.J., Francioli L.C., Tiao G., Cummings B.B., Alföldi J., Wang Q., Collins R.L., Laricchia K.M., Ganna A., Birnbaum D.P. (2019). Variation across 141,456 human exomes and genomes reveals the spectrum of loss-of-function intolerance across human protein-coding genes. bioRxiv.

[27] Lek M., Karczewski K.J., Minikel E.V., Samocha K.E., Banks E., Fennell T., O’Donnell-Luria A.H., Ware J.S., Hill A.J., Cummings B.B. (2016). Analysis of protein-coding genetic variation in 60,706 humans. Nature.

[28] Exome Variant Server, NHLBI GO Exome Sequencing Project (ESP), Seattle, WA. http://evs.gs.washington.edu/EVS/.

[29] Auton A., Brooks L.D., Durbin R.M., Garrison E.P., Kang H.M., Korbel J.O., Marchini J.L., McCarthy S., McVean G.A., 1000 Genomes Project Consortium (2015). A global reference for human genetic variation. Nature.

[30] Naslavsky M.S., Yamamoto G.L., de Almeida T.F., Ezquina S.A.M., Sunaga D.Y., Pho N., Bozoklian D., Sandberg T.O.M., Brito L.A., Lazar M. (2017). Exomic variants of an elderly cohort of Brazilians in the ABraOM database. Hum. Mutat..

[31] Adzhubei I., Jordan D.M., Sunyaev S.R. (2013). Predicting Functional Effect of Human Missense Mutations Using PolyPhen-2. Curr. Protoc. Hum. Genet..

[32] Kumar P., Henikoff S., Ng P.C. (2009). Predicting the effects of coding non-synonymous variants on protein function using the SIFT algorithm. Nat. Protoc..

[33] Quang D., Chen Y., Xie X. (2015). DANN: A deep learning approach for annotating the pathogenicity of genetic variants. Bioinformatics.

[34] Choi Y., Chan A.P. (2015). PROVEAN web server: A tool to predict the functional effect of amino acid substitutions and indels. Bioinformatics.

[35] Chun S., Fay J.C. (2009). Identification of deleterious mutations within three human genomes. Genome Res..

[36] Schwarz J.M., Cooper D.N., Schuelke M., Seelow D. (2014). MutationTaster2: Mutation prediction for the deep-sequencing age. Nat. Methods.

[37] Reva B., Antipin Y., Sander C. (2011). Predicting the functional impact of protein mutations: Application to cancer genomics. Nucleic Acids Res..

[38] Shihab H.A., Gough J., Cooper D.N., Stenson P.D., Barker G.L.A., Edwards K.J., Day I.N.M., Gaunt T.R. (2013). Predicting the Functional, Molecular, and Phenotypic Consequences of Amino Acid Substitutions using Hidden Markov Models. Hum. Mutat..

[39] Shihab H.A., Rogers M.F., Gough J., Mort M., Cooper D.N., Day I.N.M., Gaunt T.R., Campbell C. (2015). An integrative approach to predicting the functional effects of non-coding and coding sequence variation. Bioinformatics.

[40] Jagadeesh K.A., Wenger A.M., Berger M.J., Guturu H., Stenson P.D., Cooper D.N., Bernstein J.A., Bejerano G. (2016). M-CAP eliminates a majority of variants of uncertain significance in clinical exomes at high sensitivity. Nat. Genet..

[41] Kircher M., Witten D.M., Jain P., O’roak B.J., Cooper G.M., Shendure J. (2014). A general framework for estimating the relative pathogenicity of human genetic variants. Nat. Genet..

[42] Kim S., Jhong J.-H., Lee J., Koo J.-Y. (2017). Meta-analytic support vector machine for integrating multiple omics data. BioData Min..

[43] Dong C., Wei P., Jian X., Gibbs R., Boerwinkle E., Wang K., Liu X. (2015). Comparison and integration of deleteriousness prediction methods for nonsynonymous SNVs in whole exome sequencing studies. Hum. Mol. Genet..

[44] Siepel A., Bejerano G., Pedersen J.S., Hinrichs A.S., Hou M., Rosenbloom K., Clawson H., Spieth J., Hillier L.W., Richards S. (2005). Evolutionarily conserved elements in vertebrate, insect, worm, and yeast genomes. Genome Res..

[45] Cooper G.M., Stone E.A., Asimenos G., Green E.D., Batzoglou S., Sidow A., NISC Comparative Sequencing Program (2005). Distribution and intensity of constraint in mammalian genomic sequence. Genome Res..

[46] Porto F., Jones E., Branch J., Soens Z., Maia I., Sena I., Sampaio S., Simões R., Chen R. (2017). Molecular Screening of 43 Brazilian Families Diagnosed with Leber Congenital Amaurosis or Early-Onset Severe Retinal Dystrophy. Genes.

[47] Kopanos C., Tsiolkas V., Kouris A., Chapple C.E., Aguilera M.A., Meyer R., Massouras A. (2018). VarSome: The Human Genomic Variant Search Engine. bioRxiv.

[48] Flannery J.G. (1999). Transgenic Animal Models for the Study of Inherited Retinal Dystrophies. ILAR J..

[49] Veleri S., Lazar C.H., Chang B., Sieving P.A., Banin E., Swaroop A. (2015). Biology and therapy of inherited retinal degenerative disease: Insights from mouse models. Dis. Model. Mech. Mech..

[50] Petersen-Jones S.M., Komáromy A.M. (2015). Dog Models for Blinding Inherited Retinal Dystrophies. Hum. Gene Ther. Clin. Dev..

[51] Slijkerman R.W.N., Song F., Astuti G.D.N., Huynen M.A., van Wijk E., Stieger K., Collin R.W.J. (2015). The pros and cons of vertebrate animal models for functional and therapeutic research on inherited retinal dystrophies. Prog. Retin. Eye Res..

[52] Katz M.L., Redmond T.M. (2001). Effect of Rpe65 knockout on accumulation of lipofuscin fluorophores in the retinal pigment epithelium. Investig. Ophthalmol. Vis. Sci..

[53] Veske A., Nilsson S.E.G., Narfström K., Gal A. (1999). Retinal Dystrophy of Swedish Briard/Briard–Beagle Dogs Is Due to a 4-bp Deletion inRPE65. Genomics.

[54] Znoiko S.L., Rohrer B., Lu K., Lohr H.R., Crouch R.K., Ma J.-X. (2005). Downregulation of cone-specific gene expression and degeneration of cone photoreceptors in the Rpe65-/- mouse at early ages. Investig. Ophthalmol. Vis. Sci..

[55] Samardzija M., von Lintig J., Tanimoto N., Oberhauser V., Thiersch M., Remé C.E., Seeliger M., Grimm C., Wenzel A. (2008). R91W mutation in Rpe65 leads to milder early-onset retinal dystrophy due to the generation of low levels of 11-cis-retinal. Hum. Mol. Genet..

[56] Li Y., Yu S., Duncan T., Li Y., Liu P., Gene E., Cortes-Pena Y., Qian H., Dong L., Redmond T.M. (2015). Mouse model of human RPE65 P25L hypomorph resembles wild type under normal light rearing but is fully resistant to acute light damage. Hum. Mol. Genet..

[57] Choi E.H., Suh S., Sander C.L., Hernandez C.J.O., Bulman E.R., Khadka N., Dong Z., Shi W., Palczewski K., Kiser P.D. (2018). Insights into the pathogenesis of dominant retinitis pigmentosa associated with a D477G mutation in RPE65. Hum. Mol. Genet..

[58] Wenzel A., Reme C.E., Williams T.P., Hafezi F., Grimm C. (2001). The Rpe65 Leu450Met variation increases retinal resistance against light-induced degeneration by slowing rhodopsin regeneration. J. Neurosci..

[59] Wenzel A., Grimm C., Samardzija M., Remé C.E. (2003). The genetic modifier Rpe65Leu(450): Effect on light damage susceptibility in c-Fos-deficient mice. Investig. Ophthalmol. Vis. Sci..

[60] Silva E., Dharmaraj S., Li Y.Y., Pina A.L., Carter R.C., Loyer M., Traboulsi E., Theodossiadis G., Koenekoop R., Sundin O. (2004). A missense mutation in GUCY2D acts as a genetic modifier in RPE65-related Leber Congenital Amaurosis. Ophthalmic Genet..

[61] Samardzija M., Wenzel A., Naash M., Remé C.E., Grimm C. (2006). *Rpe65* as a modifier gene for inherited retinal degeneration. Eur. J. Neurosci..

[62] Narfström K., Vaegan, Katz M., Bragadottir R., Rakoczy E.P., Seeliger M. (2005). Assessment of structure and function over a 3-year period after gene transfer in RPE65-/- dogs. Doc. Ophthalmol..

[63] Nusinowitz S., Ridder W.H., Pang J.J., Chang B., Noorwez S.M., Kaushal S., Hauswirth W.W., Heckenlively J.R. (2006). Cortical visual function in the rd12 mouse model of Leber Congenital Amarousis (LCA) after gene replacement therapy to restore retinal function. Vis. Res..

[64] Aguirre G.K., Komáromy A.M., Cideciyan A.V., Brainard D.H., Aleman T.S., Roman A.J., Avants B.B., Gee J.C., Korczykowski M., Hauswirth W.W. (2007). Canine and human visual cortex intact and responsive despite early retinal blindness from RPE65 mutation. PLoS Med..

[65] Petersen-Jones S.M., Annear M.J., Bartoe J.T., Mowat F.M., Barker S.E., Smith A.J., Bainbridge J.W., Ali R.R., LaVail M., Ash J., Anderson R., Hollyfield J., Grimm C. (2012). Gene Augmentation Trials Using the Rpe65-Deficient Dog: Contributions Towards Development and Refinement of Human Clinical Trials. Retinal Degenerative Diseases. Advances in Experimental Medicine and Biology.

[66] Zheng Q., Ren Y., Tzekov R., Zhang Y., Chen B., Hou J., Zhao C., Zhu J., Zhang Y., Dai X. (2012). Differential proteomics and functional research following gene therapy in a mouse model of Leber congenital amaurosis. PLoS ONE.

[67] Jo D.H., Song D.W., Cho C.S., Kim U.G., Lee K.J., Lee K., Park S.W., Kim D., Kim J.H., Kim J.-S. (2019). CRISPR-Cas9-mediated therapeutic editing of Rpe65 ameliorates the disease phenotypes in a mouse model of Leber congenital amaurosis. Sci. Adv..

[68] Takahashi Y., Moiseyev G., Ma J. (2014). Identification of key residues determining isomerohydrolase activity of human RPE65. J. Biol. Chem..

[69] Li G., Gao G., Wang P., Song X., Xu P., Xie B., Zhou T., Pan G., Peng F., Zhang Q. (2019). Generation and Characterization of Induced Pluripotent Stem Cells and Retinal Organoids From a Leber’s Congenital Amaurosis Patient With Novel RPE65 Mutations. Front. Mol. Neurosci..

[70] Yang U., Gentleman S., Gai X., Gorin M.B., Borchert M.S., Lee T.C., Villanueva A., Koenekoop R., Maguire A.M., Bennett J. (2019). Utility of In Vitro Mutagenesis of RPE65 Protein for Verification of Mutational Pathogenicity Before Gene Therapy. JAMA Ophthalmol..

