# Supplementary material for: Pathogenicity Reclassification of RPE65 Missense Variants Related to Leber Congenital Amaurosis and Early-Onset Retinal Dystrophy
Source: Genes (Basel). 2019 Dec 24;11(1):24. doi: 10.3390/genes11010024 (PMC7016655; doi:10.3390/genes11010024)
Supplement: Supplementary file 1 [file genes-11-00024-s001.pdf]

Supplementary material

Genes targeted on retinopathy NGS panel.

ABCA4, ABCC6, ABCD1, ABHD12, ACO2, ADAM9, ADGRV1, AHI1, AIPL1, ALMS1, AMACR, ARL13B, ARL6, ATF6, B9D1, B9D2, BBS1, BBS10, BBS12, BBS2, BBS4, BBS5, BBS7, BBS9, BEST1, C12orf65, C1QTNF5, C2orf71, C5orf42, C8orf37, CA4, CABP4, CACNA1F, CACNA2D4, CASK, CC2D2A, CDH23, CDH3, CDHR1, CEP290, CEP41, CERKL, CFH, CHM, CIB2, CISD2, CLN3, CLN5, CLN6, CLN8, CLRN1, CNGA1, CNGB1, CNGB3, CNNM4, CRB1, CRX, CTSD, CYP4V2, DHDDS, DNAJC5, EFEMP1, ELOVL4, EYS, FAM161A, FLVCR1, FRMD7, FSCN2, FZD4, GDF6, GJB2, GJB6, GNAT1, GNAT2, GNPTG, GPR143, GPR179, GRK1, GRM6, GRN, GUCA1A, GUCA1B, GUCY2D, HARS, HGSNAT, HK1, HMCN1, HMX1, IDH3B, IFT140, IMPDH1, IMPG2, IQCB1, ITM2B, KCNJ13, KCNV2, KCTD7, KIF7, KLHL7, LAMA1, LCA5, LRAT, LRP5, LZTFL1, MAK, MERTK, MFN2, MFRP, MFSD8, MKKS, MKS1, MMACHC, MVK, MYO7A, NDP, NEUROD1, NMNAT1, NPHP1, NPHP3, NPHP4, NR2F1, NRL, NYX, OAT, OFD1, OPA1, OPA3, OPN1LW, OPN1MW, OTX2, PAX6, PCDH15, PDE6A, PDE6B, PDE6C, PDE6G, PDE6H, PDZD7, PEX1, PEX10, PEX11B, PEX12, PEX13, PEX14, PEX16, PEX19, PEX2, PEX26, PEX3, PEX5, PEX6, PEX7, PHYH, PITPNM3, PNPLA6, PPT1, PRCD, PROM1, PRPF3, PRPF31, PRPF6, PRPF8, PRPH2, PRPS1, RAB28, RAX2, RBP3, RBP4, RD3, RDH12, RDH5, RGR, RGS9, RGS9BP, RHO, RIMS1, RLBP1, ROM1, RP1, RP1L1, RP2, RP9, RPE65, RPGR, RPGRIP1, RPGRIP1L, RS1, SAG, SDCCAG8, SEMA4A, SLC24A1, SNRNP200, SPATA7, TCTN1, TCTN2, TEAD1, TIMM8A, TIMP3, TMEM126A, TMEM138, TMEM216, TMEM237, TMEM67, TOPORS, TPP1, TRIM32, TRPM1, TSPAN12, TTC21B, TTC8, TUBGCP4, TUBGCP6, TULP1, TYR, UNC119, USH1C, USH1G, USH2A, VPS13B, WDPCP, WDR19, WFS1, WHRN, and *ZNF513*
